# Skin Exposure to Narrow Band Ultraviolet (UVB) Light Modulates the Human Intestinal Microbiome

**DOI:** 10.3389/fmicb.2019.02410

**Published:** 2019-10-24

**Authors:** Else S. Bosman, Arianne Y. Albert, Harvey Lui, Jan P. Dutz, Bruce A. Vallance

**Affiliations:** ^1^Department of Pediatrics, BC Children’s Hospital Research Institute, University of British Columbia, Vancouver, BC, Canada; ^2^BC Women’s Hospital and Health Centre, Women’s Health Research Institute, Vancouver, BC, Canada; ^3^Department of Obstetrics and Gynaecology, University of British Columbia, Vancouver, BC, Canada; ^4^Department of Dermatology and Skin Science, University of British Columbia, Vancouver, BC, Canada; ^5^British Columbia Cancer Agency, Departments of Cancer Control Research and Integrative Oncology, Vancouver, BC, Canada; ^6^BC Children’s Hospital Research Institute, University of British Columbia, Vancouver, BC, Canada

**Keywords:** microbiome, UVB light, phototherapy, vitamin D, diversity, 16S

## Abstract

The recent worldwide rise in idiopathic immune and inflammatory diseases such as multiple sclerosis (MS) and inflammatory bowel diseases (IBD) has been linked to Western society-based changes in lifestyle and environment. These include decreased exposure to sunlight/UVB light and subsequent impairment in the production of vitamin D, as well as dysbiotic changes in the makeup of the gut microbiome. Despite their association, it is unclear if there are any direct links between UVB light and the gut microbiome. In this study we investigated whether exposing the skin to Narrow Band Ultraviolet B (NB-UVB) light to increase serum vitamin D levels would also modulate the makeup of the human intestinal microbiota. The effects of NB-UVB light were studied in a clinical pilot study using a healthy human female cohort (*n* = 21). Participants were divided into those that took vitamin D supplements throughout the winter prior to the start of the study (VDS+) and those who did not (VDS−). After three NB-UVB light exposures within the same week, the serum 25(OH)D levels of participants increased on average 7.3 nmol/L. The serum response was negatively correlated to the starting 25-hydroxy vitamin D [25(OH)D] serum concentration. Fecal microbiota composition analysis using 16S rRNA sequencing showed that exposure to NB-UVB significantly increased alpha and beta diversity in the VDS− group whereas there were no changes in the VDS+ group. Bacteria from several families were enriched in the VDS− group after the UVB exposures according to a Linear Discriminant Analysis (LDA) prediction, including *Lachnospiracheae, Rikenellaceae, Desulfobacteraceae, Clostridiales vadinBB60 group, Clostridia* Family *XIII, Coriobacteriaceae, Marinifilaceae*, and *Ruminococcus.* The serum 25(OH)D concentrations showed a correlation with the relative abundance of the *Lachnospiraceae*, specifically members of the *Lachnopsira* and *Fusicatenibacter* genera. This is the first study to show that humans with low 25(OH)D serum levels display overt changes in their intestinal microbiome in response to NB-UVB skin exposure and increases in 25(OH)D levels, suggesting the existence of a novel skin-gut axis that could be used to promote intestinal homeostasis and health.

**Clinical Trial Registration:** clinicaltrials.gov, NCT03962673. Registered 23 May 2019 – Retrospectively registered, https://clinicaltrials.gov/ct2/show/NCT03962673?term=NCT03962673&rank=1.

## Introduction

Chronic inflammatory diseases such as multiple sclerosis (MS) and inflammatory bowel diseases (IBD) have become a scourge of developed Western countries, while the incidence of these diseases is also increasing in developing countries. The exact etiology of most chronic inflammatory diseases is largely unknown but likely reflects maladaptive interactions between genetic predisposition and environmental factors that drive disease pathogenesis. These environmental factors include limited exposure to sunlight (UVB) resulting in reduced serum 25-hydroxy vitamin D (25(OH)D) levels, as well as a Westernized diet and widespread antibiotic use (ter Horst et al., 2016; Celiberto et al., 2018). Notably, the last two factors are thought to exert their effects by influencing the makeup and function of the intestinal microbiome. Dysbiosis of the gut microbiome composition and function is commonly seen in patients with chronic inflammatory diseases, with loss of beneficial microbes, as well as their metabolic products (El Aidy et al., 2012) postulated to facilitate disease. Clearly, defining the basis for these changes, as well as identifying new ways to promote a beneficial microbiome will be essential to maintaining overall health.

Vitamin D is another environmental factor known to promote intestinal health. The lipid-soluble vitamin enhances intestinal barrier integrity by promoting the expression of genes that are under transcriptional regulation of the vitamin D receptor (VDR), including those encoding tight junction proteins, autophagy related factors, and antimicrobial peptides (Kong et al., 2007; Liu et al., 2013; Assa et al., 2014; Sun, 2015). Also, innate and adaptive immune cells are influenced by vitamin D, with the vitamin suppressing pro-inflammatory responses (Clark and Mach, 2016). Vitamin D deficiency has been shown to promote an inflammatory environment which leads to dysbiosis of the gut microbiota, even in clinically healthy individuals (Luthold et al., 2017). Oral vitamin D supplementation is known to be beneficial for individuals who suffer from chronic inflammatory diseases (Garg et al., 2017; Kanhere et al., 2018). Oral supplementation of individuals suffering vitamin D deficiency was also found to have a taxa specific effect on their microbial composition, correlating with their serum levels of 25(OH)D. Supplementation led to an inverse relationship between *Ruminococcus* and *Proteobacteria* abundance and an overall increase in *Bacteroidetes* abundance in their fecal microbiome composition (Ciubotaru et al., 2016; Luthold et al., 2017; Waterhouse et al., 2018). Similarly, the lack of the VDR or vitamin D metabolism in mice leads to an altered microbiome and increased susceptibility to chemically induced models of colitis (Ooi et al., 2013). It is thought that the microbial composition is not directly shaped through vitamin D, but rather by the gene products under transcriptional control of the VDR (Cantorna et al., 2014).

The limited availability of vitamin D from dietary sources ensures that in humans, 80% of their vitamin D requirements need to be met through exposure to Ultraviolet B (UVB) light (Holick, 2002; Patwardhan et al., 2018). UVB light (light emitted wavelengths between 280 and 315 nm) produces vitamin D in the skin through the conversion of the cholesterol derivative 7-dehydrocholesterol, resulting in an increase in serum levels of 25(OH)D, the biological inactive form of vitamin D. Sun avoidance, dwelling far away from the equator, and lifestyle are some of the factors that limit exposure to UVB light on a regular basis (Lu et al., 2015). These factors contribute to the high rates of vitamin D insufficiency or deficiency seen worldwide, especially in locations with marked seasonality where the lack of UVB available from sunlight during the winter months prevents vitamin D production. Limited UVB exposure is one of the most important environmental factors linked to the onset of immune mediated chronic inflammatory diseases, like IBD and MS (Nerich et al., 2011; Breuer et al., 2014; Lu et al., 2015; Vernia et al., 2017). A reverse correlation between latitude and disease severity/prevalence is seen for these conditions, especially in northern countries that display high rates of chronic inflammatory diseases. Studies support the concept that UVB light is beneficial to health, beyond its ability to promote cutaneous vitamin D production (Grant, 2016; Langer-Gould et al., 2018). The local beneficial effects of UVB light exposure have been extensively examined for various dermatoses (Shintani et al., 2008; Rácz et al., 2011; Iyama et al., 2014), however, the potential for UVB light to affect the intestinal mucosal immune system and the gut microbiota has received little attention. Moreover, it is unclear whether such changes would be dependent or independent of the effects of UVB light on systemic levels of vitamin D.

In this study we investigated whether repeated exposure of the skin to NB-UVB light would alter the gut microbiota composition of healthy female volunteers in a clinical pilot study. We found a significant effect on the microbiota composition after repeated exposures, specifically for subjects that were not taking vitamin D supplements and thus suffering vitamin D insufficiency prior to the study. Both the alpha and beta diversity of their microbiota improved in response to UVB light exposure. The differential abundance in the participants showed an enrichment in several genera from the *Lachnospiraceae, Ruminococcus*, and *Clostridiaeae* families after the UVB light exposures. The results from this study suggest that skin exposure to NB-UVB light can exert distinct regulatory effects on the intestinal microenvironment of humans, with potential health benefits.

## Materials and Methods

### Participants Pilot Study

Healthy Caucasian females between ages 19 and 40 were recruited for the study. Only participants with Fitzpatrick Skin Types I–III were included in the study. Skin type was confirmed with a questionnaire about past tanning behavior and previous responses to sun exposure (Trakatelli et al., 2017). Individuals were excluded from participation if they had visited a sunny destination outside Canada during the 3 months prior to the study date or reported any medication-induced sun sensitivity. Participants were asked to avoid any major dietary changes from their usual diet during their participation in the study. Participants were also queried if they were taking oral vitamin D supplements, and if so, the supplement doses were recorded. The study was performed during February–April 2018 in the city of Vancouver, BC (49°N) and there was no significant ambient UVB intensity measured during the study period. Ethics for this study were approved and obtained from the UBC Research Ethics Board under protocol number H17-00303.

### UVB Light Exposures

Participants visited the UBC Skin Care Clinic and were exposed to Narrow Band UVB light (NB-UVB) (peak emission at 311 nm) in a Houva II high output phototherapy device equipped with 48 NB-UVB bulbs (Philips TL01, Eindhoven, The Netherlands). The device was calibrated each morning to correct for irradiance fluctuations. Participants underwent three full-body exposures within the same week while wearing underwear and protective UVB-blocking goggles. The starting dose was calculated for each Fitzpatrick skin type as 70% of the minimal erythemal dose (MED) as tolerated in the Skin Care Clinic: 105 mJ/cm^2^ for skin type I, 140 mJ/cm^2^ for skin type II, and 168 mJ/cm^2^ for skin type III. The dose was increased by 20% for the subsequent two visits except when the participant reported any adverse skin reactions like redness or itchiness. Two participants with Fitzpatrick skin type III did not receive increasing UVB dosages during the third visit because they displayed a mild erythemal reaction in response to their second UVB exposure.

### Sample Collection

Participants traveled to the nearest Life Labs^®^ Medical Laboratory Services location where their peripheral blood was collected for standard serum 25(OH)D analysis, up to 2 days prior to the start of the UVB light exposures and a second blood sample was taken at least 24 h after the third exposure. The vitamin D status of the participants was determined by measuring the combined serum concentrations of 25(OH)D_2_ and 25(OH)D_3_ by LC-MS/MS methods as per routine protocols of Life Labs Medical Laboratory Services.

Two separate fecal samples were collected during the 3 days before the study with OMNIGene^®^ gut stool collection kits (Genotek) and an additional two fecal samples were collected in the 3 days following the third UVB light exposure. After their collection, samples were immediately immersed in DNA stabilizing reagent and stored in the freezer. All four samples were collected from the participant at the same time to minimize freeze-thaw cycles. Samples were aliquoted and stored at −80°C until analysis.

### Microbiota Analysis

The fecal material was stored at −80°C prior to DNA extraction with the MO BIO PowerFecal DNA Kit. Amplicon fragments for the library were generated with the high-fidelity Phusion polymerase in a PCR reaction using 16S V6-V8 fusion primers (Comeau et al., 2011). Library quality was verified by using a Hamilton Nimbus Select robot to run a Coastal Genomics Analytical gel. Multiplex products were cleaned and normalized using the Charm Biotech Normalization Kit prior to the sequencing reaction using Illumina MiSeq chemistry. DNA extraction and sequencing were performed by the Integrated Microbiome Resource (Dalhousie University, Halifax, NS, Canada). Subsequent analysis was done using the Microbiome Helper pipeline (Comeau et al., 2017). Reads were trimmed with Cutadapt, paired-end reads were joined with VSEARCH and filtered out with QIIME2. Sequences were denoised with Deblur by filtering out singletons. Taxonomy was assigned using the Silva database (Quast et al., 2012). Microbiome statistical analysis was done using R studio with the Phyloseq v1.24.2 and Microbiome v3.8 (Shetty et al., 2017) packages available through Bioconductor. Visualization was done with ggplot2 v3.1.0. Relative abundance calculations were done after center log transformation with ALDEx2 v1.12.0 and Vegan v2.5-3 (Quinn et al., 2018). Linear discriminant analysis (LDA) effect size (LEfSe) algorithm was performed with the Galaxy software package of the Huttenhower lab to identify differential abundances of microbes in the UVB treated samples (Segata et al., 2011).

### Statistical Analysis

Statistical analysis and representation were performed using the GraphPad Prism software (GraphPad Software, San Diego, CA, United States) or with the use of statistical packages in R. Significance of non-parametric data was calculated using a Mann Whitney *t-*test or a one-way ANOVA with a Kruskal-Wallis *post hoc* test. Significant results are depicted in the figures (^∗^*p* < 0.05, ^∗∗^*p* < 0.001, ^∗∗∗^*p* < 0.0001).

## Results

### Participant Characteristics

Participants enrolled in the study were all females with a mean age of 28.43 years (SEM 0.85), and the study was conducted during February-April 2018 in Vancouver, Canada (latitude 49°N). No UVB light from ambient sun exposure was detected while the study was running. A total of 23 participants were enrolled in the study. The participants were self-identified healthy and were not exposed to sunlight outside of Canada during the 3 months prior to the study, either through exposure to ambient UVB light or by using a tanning bed. By the end of the study period, 2 participants were excluded from the cohort because they failed to meet the study requirements during the sample collection phase (Supplementary Figure S1). Out of the remaining 21 participants, 12 were determined to have Fitzpatrick skin type III (57.1%), 8 participants had skin type II (38.1%) and one participant had skin type I (4.8%) (Table 1).

**TABLE 1:** Baseline characteristics of study participants.

**Characteristic**	**All participants**	**VDS−**	**VDS+**
Age, years (SEM)	28.43 (0.85)	27 (0.93)	30.33 (1.36)
Participants	21	12 (57.1%)	9 (42.9%)
Fitzpatrick skin type			
Skin type 1	1 (4.8%)	0	1 (11.1%)
Skin type 2	8 (38.1%)	6 (50%)	2 (22.2%)
Skin type 3	12 (57.1%)	6 (50%)	6 (66.6%)

### Vitamin D Supplementation Increases Serum 25-OH Vitamin D Levels

**FIGURE 1:**
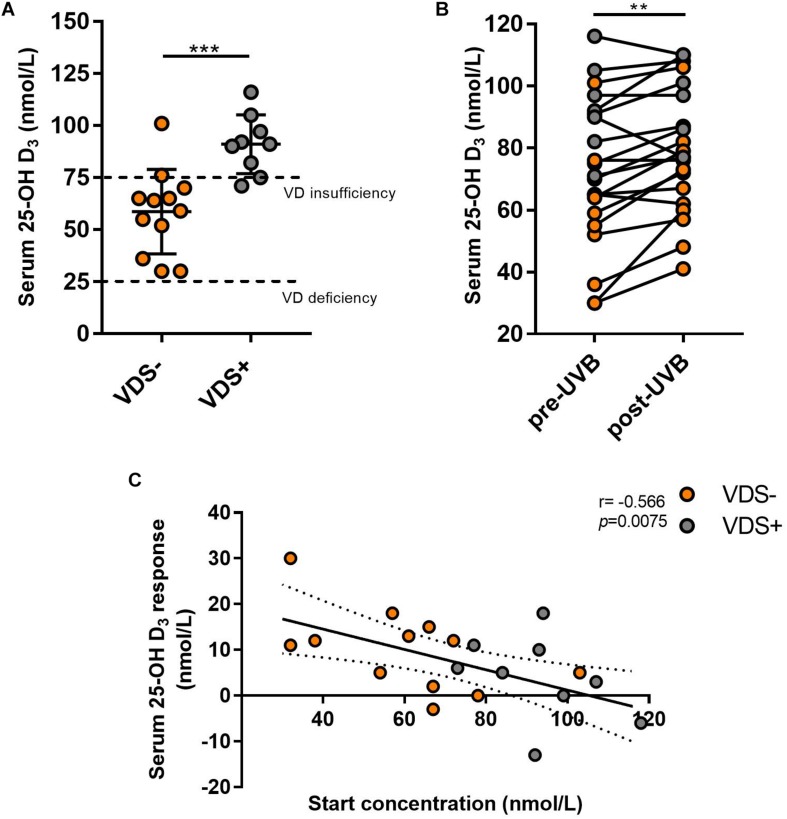
Serum 25-(OH)D response after UVB light exposures. **(A)** The serum concentrations from the VDS− group was significantly lower than the VDS + group prior to the UVB light exposures (unpaired *t*-test *p* = 0006). **(B)** Serum 25(OH)D concentration values pre and post-UVB exposures, significance was calculated using a paired *t*-test of repeated measures (*p* = 0.002). **(C)** The correlation between the serum 25(OH)D response and the 25(OH)D at the start of the study. A negative correlation was calculated with a Pearson correlation analysis (*r* = −0.566; *p* = 0.0075). ^∗∗^*p* < 0.001, ^∗∗∗^*p* < 0.0001.

Out of the 21 participants, 9 (42%) indicated they had taken vitamin D supplements during the past 3 months. The oral vitamin D supplementation doses taken by these participants ranged between 500 IU and 3500 IU/day, with an average of 1389 IU/day (6 taking 1000 IU/day). Participants reporting they had taken vitamin D supplements before the study were considered the VDS+ group while the remaining 12 participants that had not taken such supplements were considered the VDS− group. Based on their serum 25(OH)D analysis, it appeared that most participants from the VDS+ group carried vitamin D serum levels (>75 nmol/L) that would be characterized as vitamin D sufficient, whereas the majority of the VDS− group displayed lower vitamin D serum levels in the insufficient range (between 25 and 75 nmol/L), except for one outlier whose serum 25(OH)D concentration was 116 nmol/L (Figure 1A). These findings indicate that vitamin D supplementation is necessary to maintain adequate serum vitamin D levels during the winter months in a region where there is no year-round ambient UVB light available.

Serum analysis before and after UVB light exposures showed a significant increase in 25(OH)D concentrations across all participants with a mean average of 7.3 nmol/L (*p* = 0.002, mean ± SEM = 2.06), which reflects an average 10.1% increase in serum 25(OH)D levels (Figure 1B). Surprisingly, some of the participants showed no induction in serum 25(OH)D levels and two participants showed a decrease in serum 25(OH)D concentrations. The 25(OH)D serum response was similar between the two major Fitzpatrick skin types (type II and III) (*p* = 0.207), indicating that the adjusted NB-UVB dosages used for the solar sensitivity of the different skin types were effective at inducing production of vitamin D. The VDS− group showed a greater (albeit not significant) serum response (10.1 nmol/L, SEM ± 2.6) than that observed for the VDS+ group (3.8 nmol/L, SEM ± 3.1), with the participants with the lowest starting concentrations showing the highest 25(OH)D serum response (Figure 1C). A negative correlation was calculated between the 25(OH)D serum starting concentration and the participants 25(OH)D serum response (Spearman coefficient *r* = −0.566, *p* = 0.008).

### Microbiota Composition After the UVB Exposures

**FIGURE 2:**
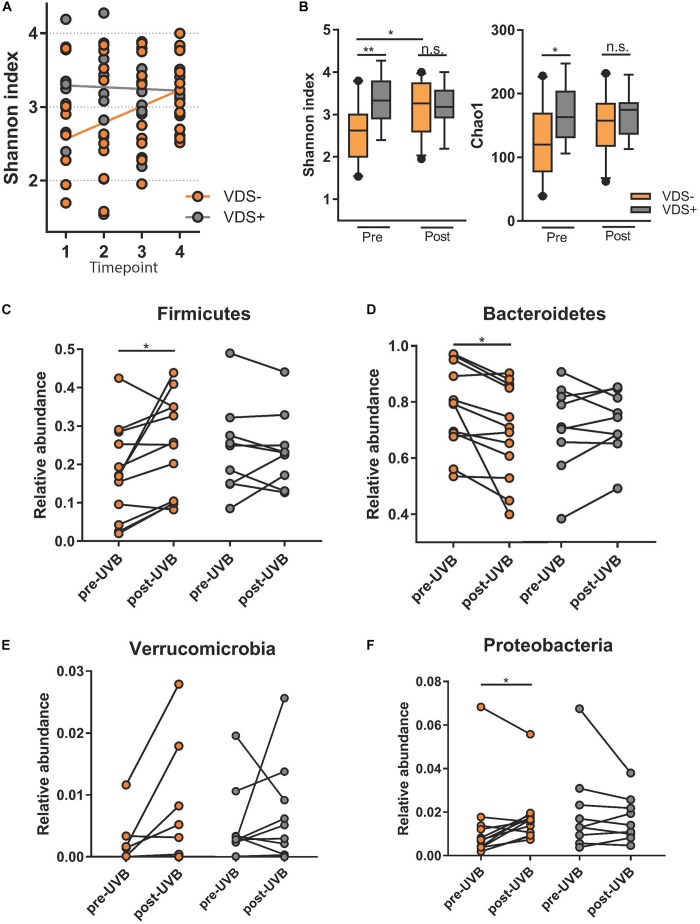
Microbiome analysis comparing study cohort before and after UVB exposures. Time points 1 and 2 are pre-UVB exposure and timepoints 3 and 4 are after UVB exposures. **(A)** Linear mixed model of the Shannon index of the different timepoints shows a significant effect of time for the UVB light exposures in the VDS− group, but not the VDS + group (*p* = 0.0001). **(B)** Alpha diversity measures diversity and richness before and after the UVB light exposures of the supplementation groups, and shows a significant difference before the UVB exposures, but not after the UVB exposures. An increase in diversity was observed only for the VDS− group (multiple comparison one-way ANOVA, *p-*adj 0.024). **(C)** Comparative analysis of taxa specific relative abundance of *Firmicutes* pre and post-UVB light exposure. Each dot represents the average relative abundance of the two microbiome samples per timepoint from each participant. A significant increase in *Firmicutes* was found for the VDS− group (*p* = 0.0287), but no significant difference in the VDS + group. **(D)** A significant decrease of *Bacteroidetes* was found for the VDS− group (*p* = 0.0164). **(E)** No significant differences of *Verrucomicrobia* was detectable pre and post UVB light exposures for both VDS groups**. (F)** A significant increase in the relative abundance of *Proteobacteria* was found for the VDS− group (*p* = 0.0493), while no significant difference was found in the VDS + group. ^∗^*p* < 0.05, ^∗∗^*p* < 0.001.

To test whether the exposure to UVB light influenced the composition of their intestinal microbiome, each participant collected four stool samples. Samples at timepoints 1 and 2 were collected during the 3 days just prior to the UVB exposures and timepoints 3 and 4 were collected in the 3 days after the last UVB exposure. The fecal microbiota composition was analyzed by sequencing of the V6-V8 hypervariable regions of the 16S rRNA gene using the MiSeq Illumina platform, using Qiime2 pipelines for analysis. First, we tested for the effect of time on alpha diversity measures using mixed-effects models that take into account the repeated measures for the two VDS groups (Figure 2A). A significant increase of the Shannon index was observed over time for the VDS− group where no difference was seen in the VDS+ group (*p* = 0.0001). Then, we tested for diversity differences between the VDS groups by pooling the repeated measures from before and after the UVB light exposure and compared with one-way ANOVA (Figure 2B). The VDS− group showed significantly lower diversity and richness of their fecal microbiome composition prior to UVB exposure than the VDS+ group, as assessed using the Shannon index (*p* adj. = 0.0019) and Chao1 measure (*p* adj. = 0.017), respectively. After the UVB exposures the Shannon diversity increased in the VDS− group (*p* = 0.0162), resulting in similar diversity with that of the VDS+ group post-UVB exposure. These findings suggest that otherwise healthy individuals carrying insufficient 25(OH)D serum levels have a less diverse microbiome composition as compared to individuals that are vitamin D sufficient. Also, UVB light exposures are able to increase the richness and evenness of the microbiome composition when given to VDS− individuals with a low starting microbial diversity.

The effect of the UVB light exposures on the relative abundance of four different phyla was tested for the two VDS groups. A paired analysis found that the VDS− group showed an increase in the relative abundance of *Firmicutes* (*p* = 0.0287) (Figure 2C), and a significant decrease in *Bacteroidetes* (*p* = 0.0164) (Figure 2D). No significant changes were observed for *Verrucomicrobia*, although it is noteworthy that there were four participants that showed a strong increase in this phylum after the UVB light exposures (Figure 2E). Additionally, a significant increase in *Proteobacteria* was found after the UVB light exposures in the VDS− group (*p* = 0.0493) (Figure 2F). In contrast, the VDS+ group did not show any significant differences in the relative abundance of *Proteobacteria* in response to the UVB light exposures.

**FIGURE 3:**
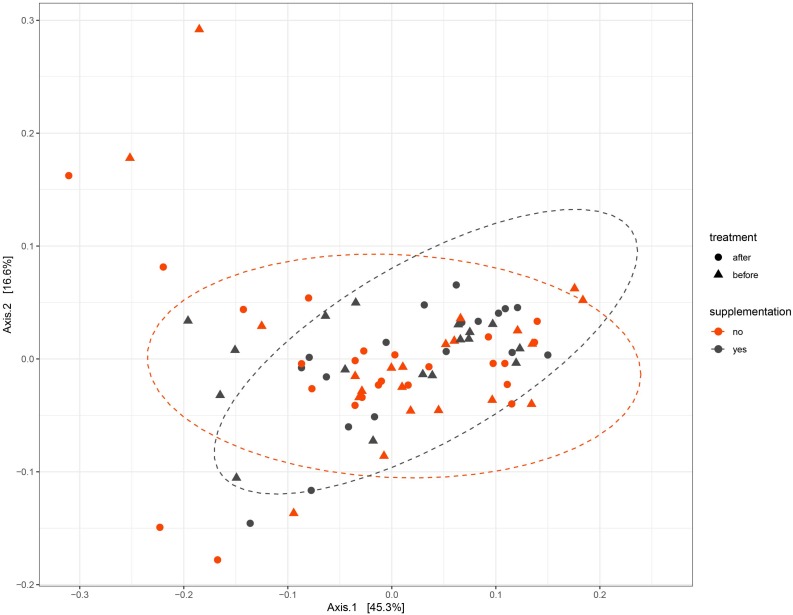
PCA of Weighted Unifrac distances. Samples from each participant cluster closer together rather than separating into pre- and post-UVB exposure. The dotted ellipses are showing the ordination direction and clusters the samples of the VDS groups, post-UVB exposure.

To test whether the UVB exposures modulated the gut microbiome composition, the beta diversity between samples was visualized with a principle component analysis using weighted Unifrac distances on centered log ratio (clr) transformed feature count data (Figure 3). The samples from each participant clustered together, meaning that the inter-personal variability was greater than the intra-personal changes that were caused by the UVB light exposures. The effect of the UVB exposures were calculated with a permutational multivariate analysis of variance (PERMANOVA) to assess the diversity between the UVB light exposures within each participant. The UVB exposures showed a significant relationship with the microbiome composition (*p* = 0.04) for the samples of the entire cohort, but no specific taxa were predicted to drive the changes in microbiota composition when tested with a LDA effect size. A PERMANOVA was performed on the two VDS groups separately. No significant differences were found after UVB exposure in the VDS+ group (*p* = 0.723), but the composition in the VDS− group was found to be significantly different after the UVB exposures *(p* = 0.001, Turkey’s HSD *p* = 0.041). Despite the small sample size of the groups, these findings suggest that UVB exposures caused a shift in the microbiome of those participants (VDS−) that were not taking vitamin D supplements prior to the study.

**FIGURE 4:**
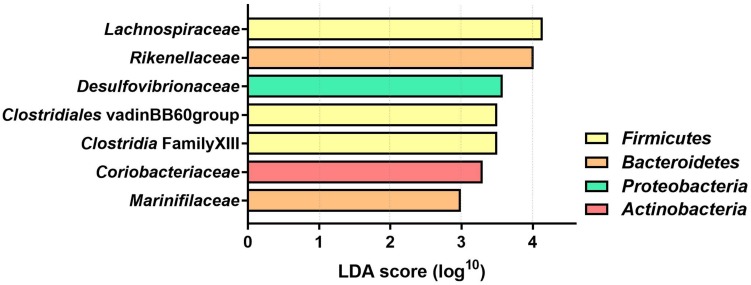
Linear Discriminant analysis (LDA) score of differentially abundant bacterial families in the VDS− group. The effect of the UVB light exposures was calculated with a two-factor PERMANOVA, controlling for the interpersonal variation before and after the UVB light exposures. Only the bacterial families with significantly different (*p* < 0.05) LDA score are depicted (*n* = 46 samples collected from 12 participants).

The LDA effect size was calculated for Family and Genus levels of the VDS− group to identify which bacterial taxa were most likely responsible for the different composition before and after the UVB light exposures (Figure 4). The largest effect was explained by an enrichment in *Lachnospiraceae* (LDA 4.14, *p* = 0.038), specifically for different taxa at genus level, including *Lachnospira* (LDA 3.308, *p* = 0.040), *Agathobacter* (LDA 2.924, *p* = 0.031), *Dorea* (LDA 2.916, *p* = 0.031), *CAG_56* (LDA 2.892, *p* = 0.031), *Fusicatenibacter* (LDA 2.654, *p* = 0.041) (Table 2). Other significantly enriched taxa in the post-UVB exposure samples were *Rikenellaceae alistipes* (LDA 4.010, *p* = 0.031), *Desulphovibrionaceae bilophila* (LDA 3.579 p = 0.031), *Clostridiales vadin* BB60 group (LDA 3.501 *p* = 0.040), *Clostridia* Family XIII AD3011 group (LDA 3.501 *p* = 0.031), *Coriobacteriaceae collinsella* (LDA 3.299 *p* = 0.039) *Marinifilaceae odoribacter* (LDA 2.992 *p* = 0.031). Two members from the *Ruminococcus* family [*C. intestimonas (LDA 3.089 p* = 0.041) *and C. ruminococcus* (LDA 2.841 *p* = 0.031)] were found to be significantly increased at the genus level but not at the family level.

**TABLE 2:** Linear effect size score (LEfSe) that predicts which genera are responsible for the differential bacterial composition after the UVB light exposure of the VDS− group.

**Phylum**	**Family**	**Genus**	**LDA score**	***P.adjust*-value**
*Firmicutes*	*Lachnos- piraceae*	*Lachnospira*	3.308	0.040
		*Agathobacter*	2.924	0.031
		*Dorea*	2.916	0.031
		*CAG-56*	2.892	0.031
		*Fusicatenibacter*	2.654	0.041
*Bacteroidetes*	*Rikenellaceae*	*Alistipes*	3.914	0.031
*Proteobacteria*	*Desulfovi- brionaceae*	*Bilophila*	2.872	0.031
*Firmicues*	*Clostridiales vadinBB60 group*	*Clostridiales*	2.739	0.040
*Firmicutes*	*Clostridia Family XIII*	*Family XIII AD3011group*	2.565	0.031
*Actinobacteria*	*Corio- bacteriaceae*	*Collinsella*	2.752	0.039
*Bacteroidetes*	*Marinifilaceae*	*Odoribacter*	2.774	0.031
*Firmicutes*	*Ruminococcus*	*Intestinimonas*	3.089	0.041
		*Ruminococcus*	2.841	0.031

**14 different bacterial genera from 7 different families were predicted to drive the separation after the UVB light exposures in the VDS− group. Adjusted p-values were calculated using Benjamin Hochberg correction method for multiple testing.**

**FIGURE 5:**
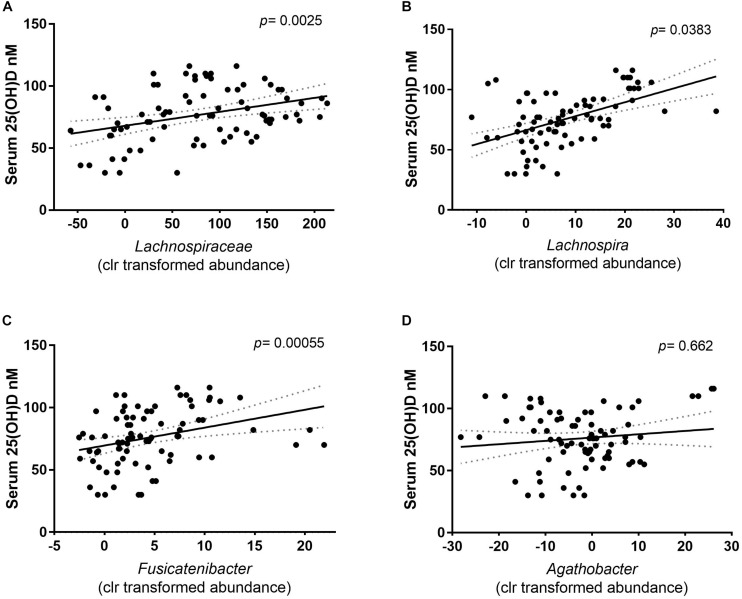
Correlation of the relative abundance (clr) and the serum 25(OH)D concentration of the participants. The solid line depicts the mean and dotted area the calculated error of the mean. Each dot depicts the centered log ratio relative abundance per sample (*n* = 81). **(A)** The association of the family *Lachnospiraceae* with serum 25(OH)D concentration showed a positive correlation. Correlation was calculated with a mixed- effect regression of abundance against the serum 25(OH)D concentration, of which the difference was tested with a Chi square distribution test (*p* = 0.0025). **(B)** At genus level, *Lachnospira* (*p* = 0.0383), and **(C)** *Fusicatenibacter* (*p* = 0.000055) clr relative abundance is positively correlated with the serum 25(OH)D. **(D)** The relative abundance of *Agathobacter* is independent of the serum 25(OH)D concentrations (*p* = 0.662).

In previous studies, the abundance of *Lachnospiraceae* has been linked to the vitamin D status of the host (Wang et al., 2019). The LDA results were confirmed using mixed effect regression models to calculate the association between the clr relative abundance and the serum 25(OH)D concentration of *Lachnospiraceae* family, including the predicted enriched genera from that family. A significant association was found where a higher abundance of *Lachnospiraceae* was correlated with higher serum 25(OH)D concentrations (*p* = 0.00425) (Figure 5A). Out of the five genera that had a positive LDA score, only the clr relative abundance of *Lachnospira* and the *Fusicatenibacter* were positively associated with the serum 25(OH)D concentrations (Figures 5B,C, respectively). While the members of the *Agathobacter* showed no association with the serum 25(OH)D concentrations (Figure 5D). This indicates that abundance of at least two genera of the *Lachnospiraceae* family within the intestinal microbiota might be directly promoted by the increase in vitamin D serum levels seen following UVB exposures.

## Discussion

The current study shows that three exposures to NB-UVB light caused an increase in serum 25(OH)D levels (7.3 nmol/L) in healthy individuals within a week. The finding that most of the VDS− group was vitamin D insufficient at the start of the study, whereas the VDS+ group was vitamin D sufficient confirms that oral vitamin D supplementation is an effective way to maintain 25(OH)D sufficiency when UVB availability is limited in the winter months. We found that the serum 25(OH)D concentrations increased approximately 10% within 1 week of UVB exposures, confirming that exposing the skin to NB-UVB light is an effective way to increase vitamin D serum levels (Osmancevic et al., 2015). It has previously been shown that the relationship between vitamin D production in the skin and UVB light exposure is not linear, but rather plateaus when serum levels reach the vitamin D sufficiency range (Jamil et al., 2018; Patwardhan et al., 2018). We found that the newly synthesized vitamin D is dependent on the preexisting serum 25(OH)D concentration. This finding confirms previous studies that report a negative feedback regulation whereby serum vitamin D regulates the availability of 7-dehydrocholesterol in the skin for vitamin D synthesis in response to UVB exposure (Bogh et al., 2009; Osmancevic et al., 2015; Zou and Porter, 2015; Jamil et al., 2018).

Intriguingly, these repeated sub-erythemal UVB skin exposures led to a significant increase in the alpha and beta diversity of the gut microbiome in participants who had not supplemented with vitamin D prior to the study (VDS−), even though both VDS groups showed a similar increase in their serum 25(OH)D levels. This finding suggests that the modulatory effects of UVB on the microbiome were associated with the 25(OH)D insufficiency status of the VDS− group. Based on the fact that UVB light has been previously shown to modulate the immune system, we hypothesize that exposing the skin to UVB light initially leads to local changes in both innate and adaptive immune cells (Clark and Mach, 2016). These cells subsequently traffic to more systemic sites, including the gut, where their release of mediators in turn shapes the composition of the gut microbiome. Since vitamin D deficiency has been previously correlated with microbial dysbiosis in both mice and humans, this study highlights the importance of maintaining vitamin D sufficiency. Correspondingly, participants in the VDS− group started the study with a significantly lower microbial alpha diversity as compared to the VDS+ group, with UVB exposures increasing their diversity to the same level as the VDS+ group. A diverse microbiome is thought to be more resilient against stressors and is seen as a hallmark of health (Human Microbiome Project Consortium, 2012).

The bacterial genera in the VDS− group that were differentially abundant are commensal bacteria that are associated with a healthy microbiome. Several of the enriched genera are within the bacterial family *Lachnospiraceae*, and have been previously reported to be associated with an improved health status as compared to the microbiome of those individuals suffering from diverse immune-mediated inflammatory diseases (Forbes et al., 2018). In addition, the abundance of two out of five *Lachnospiraceae* genera showed a correlation with the serum 25(OH)D levels of the participants, similar to previously described studies (Quast et al., 2012). Furthermore, enrichment of members of the *Lachnospiraceae, Ruminococcus* and *Rikenellaceae* families have been associated with a healthy microbiota composition in a meta-analysis of 3048 databases, as well as increased production of SCFA’s (Mancabelli et al., 2017; Schäffler et al., 2018; Jin et al., 2019). Because of the nature of 16S rRNA sequencing, it is impossible to identify species level changes from the analysis, however, multiple different bacterial families were differentially abundant, which does suggest a selective modification process in response to the UVB light exposures. Functional metabolomics or genomics could give a better insight into the metabolic potential of the microbiome changes that are attributes to good health, like the production of SCFAs and tryptophan metabolites.

To our knowledge, this is the first study that reports changes in the human gut microbiota in response to UVB light. The inclusion criteria of the current study design were very selective to reduce possible confounding factors like ethnicity, sex, and age. As described, this study recruited only female participants with the goal of reducing variability, and to increase the probability of participant enrollment. Even so, we expect that the gut microbiome response to UVB exposure is sex-independent and our results would be similar in male subjects. Moreover, to make stronger conclusions about the effect of UVB light on the microbiota on all humans, the study should be repeated in a larger cohort that includes a wider range of skin types and both sexes. While it would be interesting to test if full spectrum sunlight would have the same effect on the human gut microbiota, controlling such exposure would be challenging. Our observations support findings that humans display seasonal fluctuations in their microbiome composition, potentially coinciding with fluctuations in serum vitamin D levels throughout the year (ter Horst et al., 2016). Previous reporting of such seasonal fluctuations in the microbiome composition of humans (Davenport et al., 2014; Hisada et al., 2015) proposed that such changes were due to different food availabilities and consumption between seasons and over the year. Based on our findings that UVB light can rapidly modulate the gut microbiome without any dietary changes of the participants, it cannot be excluded that sun exposure is contributing to the seasonal variation in microbiome composition found in these studies. While seasonal variation of the microbiome might not have overt effects on healthy individuals, it could be of greater importance for people with immune dysfunction. Several chronic inflammatory diseases display seasonal patterns in the severity of disease (Watad et al., 2017). Specifically, the relapsing and remitting nature of IBD and MS are strongly associated with vitamin D levels (Munger et al., 2006; Abreu-Delgado et al., 2016; Watad et al., 2017). Exacerbations in IBD activity are commonly reported when serum vitamin D levels are low, with our data raising the question of whether these changes in disease activity could be precipitated by concurrent changes in microbiome composition.

## Data Availability Statement

The datasets generated and/or analyzed during the current study are available in the Sequence Read Archive (SRA), hosted by the National Center for Biotechnology Information (NCBI), available with accession number PRJNA539987.

## Ethics Statement

Ethics for the clinical study were approved and obtained from the UBC Research Ethics Board under protocol number H17-00303. All participants gave informed written consent prior to the start of the experimental procedures.

## Author Contributions

Experiments were designed, performed and reported by EB under the supervision of BV. Training and supervision for the clinical study were given by HL and JD to EB who coordinated the study logistics and conducted the participant visits to the Skin Care Centre. Microbiome samples were processed, sequenced and preprocessed by Andre Comeau at the integrated Microbiome research center. Subsequent statistical analysis of the microbiome data was done by EB with the help of AA. The manuscript was written by EB and BV.

## Conflict of Interest

The authors declare that the research was conducted in the absence of any commercial or financial relationships that could be construed as a potential conflict of interest.
