# Supplementary figures and images for: Skin Exposure to Narrow Band Ultraviolet (UVB) Light Modulates the Human Intestinal Microbiome
Source: Front Microbiol. 2019 Oct 24;10:2410. doi: 10.3389/fmicb.2019.02410 (PMC6821880; doi:10.3389/fmicb.2019.02410)

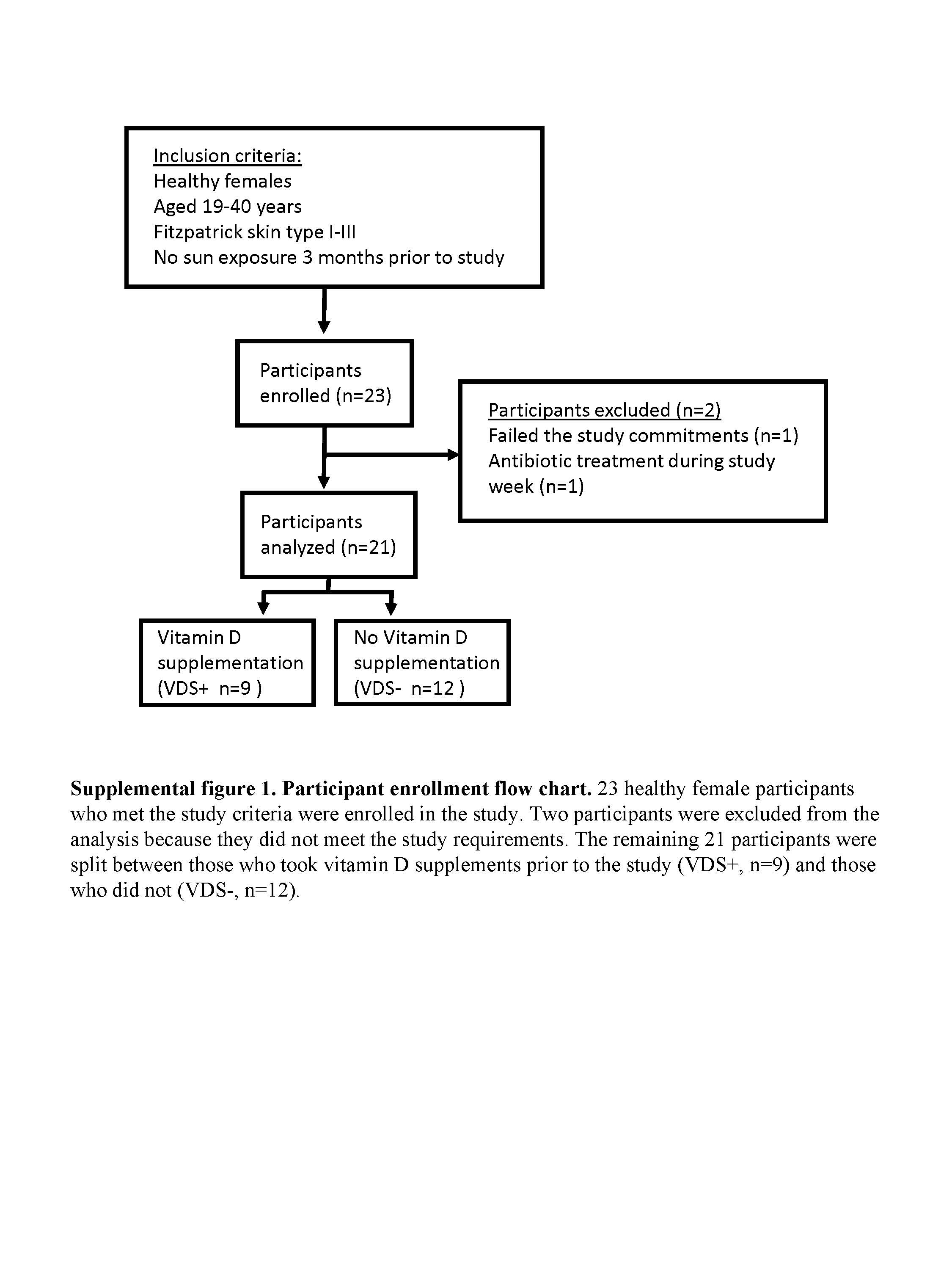

Supplement: Supplementary file 1 [file Image_1.JPEG]
